# To Will and to Do

**Published:** 1892-10-22

**Authors:** 


### Passing Topics.

### TO WILL AND TO DO.

In the last number of The Hospital the question was asked —"Where are the donors?" while a well-known witty and always welcome writer has been taking occasion to comment upon the absence of charitable bequests from the will of a recently deceased nobleman and millionaire distinguished for his amiable character. There is no gainsaying the facts, which are as clear as noon-day. The bearer of an illustrious title, the owner of large estates in half-a-dozen counties, and the possessor of nearly two millions in personalty, he might appropriately and with no appreciable inconvenience to his heirs, have apportioned a little of the vast wealth he left behind him to the cause of the poor and the afflicted, and it is strictly pertinent to inquire why has he not done so?

Well, we think one and the chief reason why persons of the highest rank and greatest means so seldom devote any portion of their wealth to philanthropic purposes is because they have never had any real knowledge of all that poverty means. It is their misfortune rather than their fault that they have ever been denied opportunities to learn what that sympathy is which is born of kindred experiences—the real, as distinguished from the merely pitiful, or to assume even for an instant the weight of the burden under which so many sink. Men and women to whom everything that money can procure has always been forthcoming, must have vivid imaginations if their sympathies are to be intelligently and comprehensively exercised on behalf of others. They cannot grasp that which for them has no real existence. Thus, they will sympathise honestly with bodily pain because at some time or another they have been the subject of it, but that does not render them any the more ready to provide the means of relief they have never known the want of. In the same way, numbers of wealthy people visit hospitals and display an amiable solicitude for the patients, as sufferers, but apparently are never conscious that the work needs money to maintain it.

As a rule, the rich are impatient, if not actually scornful of poverty. Perhaps this is because if they know anything of it at all, they only know it at its worst. They associate poverty and beggary, and they have an instinctive horror of beggars, whether they be the ragged ones solicitous of halfpence, or the well-dressed applicants for cheques. A veritable Crœsus is reported to have remarked that the only advantage he could name which the rich man possessed is that he can be robbed without feeling it. Yet the rich man seldom allows himself the enjoyment of this negative superiority. Usually, he is quick to resent the slightest attempt at extortion. It is only the poor man who can afford to exhibit equanimity under injury and over-reaching. And we are not without respect for the rich people who refuse to pay eighteenpence for an article commonly sold at a shilling. Everybody who sets his face against extortion performs a public service, and surely not the less because to submit would have caused him no inconvenience. But if the rich would only learn to understand a little better the pleasure of generosity, good work would not languish so often as it does now, and no one would begrudge them the addition to their already long list of advantages which a practical exercise of this virtue would bring them.

Agricola.

